# Long-lasting Symptoms After an Acute COVID-19 Infection and Factors Associated With Their Resolution

**DOI:** 10.1001/jamanetworkopen.2022.40985

**Published:** 2022-11-09

**Authors:** Olivier Robineau, Marie Zins, Mathilde Touvier, Emmanuel Wiernik, Cedric Lemogne, Xavier de Lamballerie, Hélène Blanché, Jean-François Deleuze, Paola Mariela Saba Villarroel, Céline Dorival, Jerome Nicol, Roselyn Gomes-Rima, Emmanuelle Correia, Mireille Coeuret-Pellicer, Nathalie Druesne-Pecollo, Younes Esseddik, Céline Ribet, Marcel Goldberg, Gianluca Severi, Fabrice Carrat

**Affiliations:** 1 Sorbonne Université, Institut National de la Santé et de la Recherche Médicale (INSERM), Institut Pierre-Louis d’Epidémiologie et de Santé Publique, Paris, France; 2 EA2694, Univ Lille, Centre Hospitalier de Tourcoing, Tourcoing, France; 3 Population-Based Epidemiological Cohorts, UMS 11, Paris-Saclay University, Versailles St Quentin University, Université de Paris, INSERM, Villejuif, France; 4 Sorbonne Paris Nord University, INSERM U1153, Inrae U1125, Conservatoire National des Arts et Metiers, Nutritional Epidemiology Research Team, Epidemiology and Statistics Research Center–University of Paris, Bobigny, France; 5 Université de Paris, INSERM U1266, Institut de Psychiatrie et Neurosciences de Paris, Paris, France; 6 Unité des Virus Emergents, UVE: Aix Marseille University, IRD 190, INSERM 1207, Institut Hospitalo-Universitaire Méditerranée Infection, Marseille, France; 7 Fondation Jean Dausset–CEPH (Centre d’Etude du Polymorphisme Humain), Paris, France; 8 Centre de recherche Epidémiologique en Santé des Populations UMR1018, Paris-Saclay University, Université de Versailles Saint-Quentin, INSERM, Gustave Roussy, Villejuif, France; 9 Department of Statistics, Computer Science, Applications “G. Parenti,” University of Florence, Florence, Italy; 10 Département de santé publique, Hôpital Saint-Antoine, Assistance Publique Hôpitaux de Paris, Paris, France

## Abstract

**Importance:**

Persistent symptoms after SARS-CoV-2 infection are an emerging public health problem. The duration of these symptoms remains poorly documented.

**Objective:**

To describe the temporal dynamics of persistent symptoms after SARS-CoV-2 infection and the factors associated with their resolution.

**Design, Setting, and Participants:**

This cross-sectional study involved 53 047 participants from 3 French adult population-based cohorts (CONSTANCES [Consultants des Centres d’Examens de Santé], E3N/E4N, and Nutrinet-Santé) who were included in a nationwide survey about SARS-CoV-2 infection. All participants were asked to complete self-administered questionnaires between April 1 and June 30, 2020. Variables included sociodemographic characteristics, comorbid conditions, COVID-19 diagnosis, and acute symptoms. Blood samples were obtained for serologic analysis between May 1 and November 30, 2020, from patients with SARS-CoV-2 infection defined as enzyme-linked immunosorbent assay immunoglobulin G antispike detection confirmed with a neutralization assay. A follow-up internet questionnaire was completed between June 1 and September 30, 2021, with details on persistent symptoms, their duration, and SARS-CoV-2 infection diagnosis by polymerase chain reaction.

**Main Outcomes and Measures:**

Persistent symptoms were defined as symptoms occurring during the acute infection and lasting 2 or more months. Survival models for interval-censored data were used to estimate symptom duration from the acute episode. Multivariable adjusted hazard ratios (HRs) were estimated for age, sex, and comorbid conditions. Factors associated with the resolution of symptoms were assessed.

**Results:**

A total of 3972 participants (2531 women [63.7%; 95% CI, 62.2%-65.2%]; mean [SD] age, 50.9 [12.7] years) had been infected with SARS-CoV-2. Of these 3972 participants, 2647 (66.6% [95% CI, 65.1%-68.1%]) reported at least 1 symptom during the acute phase. Of these 2647 participants, 861 (32.5% [95% CI, 30.8%-34.3%]) reported at least 1 persistent symptom lasting 2 or more months after the acute phase. After 1 year of follow-up, the estimated proportion of individuals with complete symptom resolution was 89.9% (95% CI, 88.7%-90.9%) with acute symptoms. Older age (>60 years; HR, 0.78; 95% CI, 0.68-0.90), female sex (HR, 0.64; 95% CI, 0.58-0.70), history of cancer (HR, 0.61; 95% CI, 0.47-0.79), history of tobacco consumption (HR, 0.80; 95% CI, 0.73-0.88), high body mass index (≥30: HR, 0.75; 95% CI, 0.63-0.89), and high number of symptoms during the acute phase (>4; HR, 0.43; 95% CI, 0.39-0.48) were associated with a slower resolution of symptoms.

**Conclusions and Relevance:**

In this cross-sectional study, persistent symptoms were still present in 10.1% of infected individuals at 1 year after SARS-CoV-2 infection. Given the high level of cumulative incidence of COVID-19, the absolute prevalent number of people with persistent symptoms is a public health concern.

## Key Points

**Question:**

What is the duration of persistent symptoms after SARS-CoV-2 infection, and what factors are associated with their resolution?

**Findings:**

This cross-sectional study nested in 3 French population-based cohorts found that approximately 10% of individuals with acute COVID-19 infection still had symptoms after 1 year of follow-up. The risk factors associated with the duration of these symptoms vary depending on their persistence.

**Meaning:**

This study suggests that persistent symptoms after SARS-CoV-2 infection is a public health concern.

## Teaser

This cross-sectional study describes the temporal dynamics of persistent symptoms after SARS-CoV-2 infection and the factors associated with their resolution.

## Introduction

Persistent symptoms after SARS-CoV-2 infection are an emerging public health problem. According to the World Health Organization (WHO), the post–COVID-19 condition, commonly referred to as long COVID, is defined as “the illness that occurs in people who have a history of probable or confirmed SARS-CoV-2 infection; usually within 3 months from the onset of COVID-19, with symptoms and effects that last for at least 2 months.”^1^ Factors such as age, female sex, comorbid conditions, and number of symptoms during the acute phase of the infection are known to be associated with the occurrence of long COVID.^2,3,4,5,6^ Whether some of these persistent symptoms will resolve more than 1 year after the acute infection has not yet been well explored, to our knowledge, especially in the general population.^7^ This work aims to describe the temporal dynamics of symptoms after SARS-CoV-2 infection, to characterize persistent symptoms, and to identify factors associated with their resolution.

## Methods

### The Survey

This cross-sectional study is part of the SAPRIS-SERO (Santé, Pratiques, Relations et Inégalités Sociales en Population Générale Pendant la Crise COVID-19–Sérologie) survey, which aims to quantify the cumulative incidence of SARS-CoV-2 infection in the French population using the dried blood spot (DBS) test for anti–SARS-CoV-2 antibodies.^8^ The SAPRIS-SERO study was approved by the Sud-Mediterranée III ethics committee, and electronic informed consent was obtained from all participants for DBS testing. This study followed the Strengthening the Reporting of Observational Studies in Epidemiology (STROBE) reporting guideline for cross-sectional studies.

In brief, data from 3 general, adult population–based French cohorts (E3N/E4N, CONSTANCES [Consultants des Centres d’Examens de Santé], and Nutrinet-Santé) were included in this study. Serologic data were collected between May 1 and November 30, 2020, to define infections that occurred during the first wave of the COVID-19 epidemic when polymerase chain reaction (PCR) testing was not available for all patients. More precisely, blood samples were obtained for serologic analysis from patients via the DBS card. Seropositive status was defined by immunoglobulin G detections against the spike protein of the virus (ie, optical density ratio ≥1.1 and <0.8) using the enzyme-linked immunosorbent assay (Euroimmun). These results were confirmed by an in-house neutralization assay to detect neutralizing anti–SARS-CoV-2 antibodies. Neutralizing antibody titers of 40 or higher were considered to be highly specific of a history of SARS-CoV-2 infection.^8^

Only participants with internet access were invited to participate in the present study. A follow-up internet questionnaire with details on clinical symptoms was completed by the participants between June 1 and September 30, 2021. Details of new symptoms since January 2020, their duration, and whether their onset was contemporaneous with an acute infectious episode were collected. Participants who did not report a symptom or a comorbid condition were considered to not have that symptom or that comorbid condition. Only individuals who participated in the first 2 waves of SAPRIS-SERO questionnaires, the serologic survey, and the follow-up questionnaire were included in the analysis. Data on the duration of symptoms were collected as intervals (<1 week, 1-2 weeks, 3-4 weeks, 5-8 weeks, >8 weeks to ≤3 months, >3 months to ≤6 months, >6 months to ≤1 year, >1 year to ≤18 months, and >18 months). The questionnaire also included information on PCR or serologic tests performed starting in January 2020.

Participants were considered to be in the study if (1) they declared a history of positive PCR test results and for whom the PCR test date was available or (2) they had a neutralization antibody titer of 40 or higher at the initial serologic analysis performed for the SAPRIS-SERO study. The acute phase of the disease was defined by the period beginning with the appearance of the first symptom associated with SARS-CoV-2 infection and ending 15 days later. A persistent symptom was defined as a symptom lasting at least 8 weeks as defined by the WHO.^9^

### Statistical Analysis

Only individuals who participated in the first 2 waves of SAPRIS-SERO questionnaires, the serologic survey, and the follow-up questionnaire were included in the analysis. Sociodemographic variables, chronic comorbid conditions prior to 2020, symptoms at the acute phase of the infection, and duration of symptoms were described. The symptom with the longest duration was used to define complete resolution of all symptoms.

We used a survival model for interval-censored data to estimate symptom duration from the acute episode. Survival time started at the onset of the symptom and was censored if the symptom was still present at the time of the follow-up questionnaire.^10^ Multivariable adjusted hazard ratios (HRs) for time to resolution of all symptoms were estimated, with 95% CIs estimated by bootstrapping with 1000 replicates (details for each symptom are presented in eTable 4 in Supplement 1).^11^ A sensitivity analysis including all symptoms declared during the follow-up period was performed using the same method. Another sensitivity analysis was performed on subgroups defined by the mode of diagnosis of SARS-CoV-2 infection (serologic test or PCR). Log-logistic distribution for survival time was found to best fit the data compared with exponential, Weibull, or gamma distributions and was used to estimate quantiles of the survival curves. Groups were compared using the *t* test for continuous variables and the χ^2^ test for qualitative variables. All tests were 2-sided, and *P* < .05 was considered statistically significant. All analyses were performed using R, version 4.0.5 (R Group for Statistical Computing) and its packages ggplot2, icenReg, and survival.^12^

## Results

Of the 56 063 individuals who participated in the first phase of the study and had interpretable serologic test results, 53 047 (94.6%) completed the follow-up questionnaire (eFigure 1 in Supplement 1). A total of 3972 participants (2531 women [63.7%; 95% CI, 62.2%-65.2%]; mean [SD] age, 50.9 [12.7] years) had SARS-CoV-2–positive biological test results. Of these 3972 participants, 3079 (77.5% [95% CI, 76.2.1%-78.8%]) underwent PCR testing, and 893 (22.5% [95% CI, 21.2%-23.8%]) underwent neutralization. Overall, 2647 of the participants with a SARS-CoV-2 infection (66.6% [95% CI, 65.1%-68.1%]) reported at least 1 symptom during the acute phase. The clinical characteristics of the participants are detailed in Table 1, and clinical characteristics by sex, age, and acute phase of the disease are detailed in eTables 1, 2, and 3, respectively, in Supplement 1. The median follow-up was 243 days (IQR, 166-465 days). Among infected individuals with symptoms during the acute phase, 861 of 2647 (32.5% [95% CI, 30.8%-34.3%]) reported at least 1 persistent symptom lasting 2 or more months after the acute phase (Table 1). The most frequent persistent symptoms were dyspnea (163 of 614 [26.5%; 95% CI, 23.1%-30.3%]), articular pain (111 of 413 [26.9%; 95% CI, 22.7%-31.5%]), anosmia or ageusia (264 of 978 [27.0%; 95% CI, 24.3%-29.9%]), asthenia (378 of 1832 [20.6%; 95% CI, 18.8%-22.6%]), attention or concentration disorders (84 of 376 [22.3%; 95% CI, 18.3%-27.0%]), memory loss (70 of 175 [40.0%; 95% CI, 32.8%-47.7%]), and sleep disorders (154 of 421 [36.6%; 95% CI, 32.0%-41.4%]) (Table 2). The estimated proportion of participants with symptoms during the acute phase who had at least 1 persistent symptom was 18.4% (95% CI, 16.5%-19.5%) at 6 months, 10.1% (95% CI, 9.1%-11.3%) at 12 months, and 7.8% (95% CI, 6.9%-8.7%) after 18 months (Table 3; eFigure 2 in Supplement 1). Among individuals with acute symptomatic infection, the estimated proportion of those who had more than 5 symptoms was 33.6% (95% CI, 31.8%-35.5%) at 1 week after the acute infection and 2.8% (95% CI, 2.2%-3.5%) at 2 months after the acute infection (Figure).

**Table 1.** Clinical Characteristics of the Population

Variable	Participants, No. (%)			*P* value
	Overall (N = 3972)	No persistent symptoms (n = 3111)	Persistent symptoms (n = 861)^a^	
Age, mean (SD), y	50.9 (12.7)	50.7 (12.8)	51.5 (12.5)	.07
Female sex	2531 (63.7)	1882 (60.5)	649 (75.4)	<.001
Chronic respiratory disease	258 (6.5)	176 (5.7)	82 (9.5)	<.001
Anxiety or depression	94 (2.4)	57 (1.8)	37 (4.3)	<.001
Cancer	163 (4.1)	108 (3.5)	55 (6.4)	<.001
Hypertension	264 (6.6)	198 (6.4)	66 (7.7)	.20
Diabetes	73 (1.8)	50 (1.6)	23 (2.7)	.06
Chronic cardiologic disorder	71 (1.8)	52 (1.7)	19 (2.2)	.37
BMI, mean (SD)	24.4 (4.3)	24.3 (4.2)	24.7 (4.7)	.03
History of tobacco consumption	1911 (48.1)	1454 (46.7)	457 (53.1)	<.003
No. of symptoms at the acute phase, mean (SD)	3.1 (3.2)	2.3 (2.7)	5.8 (3.1)	<.001

Abbreviation: BMI, body mass index (calculated as weight in kilograms divided by height in meters squared).

^a^ Persistent symptoms defined by the presence of at least 1 symptom at 2 or more months after the acute phase of the infection.

**Table 2.** Symptoms Reported During the Acute Phase of SARS-CoV-2 Infection and 2 or More Months Later (Persistent Symptoms)

Symptom	No./total No. (%) [95% CI]	
	Symptoms experienced during the acute phase (n = 3972)	Persistent symptoms (≥2 mo) among participants with symptoms at acute phase (n = 2647)
≥1 Symptom	2647/3972 (66.6) [65.1-68.1]	861/2647 (32.5) [30.7-34.3]
Cough	1091/3972 (27.5) [26.1-28.9]	81/1091 (7.4) [6.0-9.2]
Dyspnea	614/3972 (15.5) [14.4-16.6]	163/614 (26.5) [23.1-30.3]
Thoracic pain	367/3972 (9.2) [8.4-10.2]	49/367 (13.4) [10.1-17.4]
Palpitation	173/3972 (4.4) [3.8-5.0]	46/173 (26.6) [20.3-33.9]
Articular pain	413/3972 (10.4) [9.5-11.4]	111/413 (26.9) [22.7-31.5]
Myalgia	1333/3972 (33.6) [32.1-35.1]	98/1333 (7.4) [6.0-8.9]
Headache	1456/3972 (36.7) [35.2-38.2]	89/1456 (6.1) [5.0-7.5]
Cranial nerve abnormalities	7/3972 (0.2) [0.1-0.4]	2/7 (28.6) [5.1-69.7]
Sensory impairment	121/3972 (3.0) [2.5-3.6]	28/121 (23.1) [16.2-31.9]
Speech impairment	31/3972 (0.8) [0.5-1.1]	4/21 (19.0) [6.3-42.6]
Hearing impairment	58/3972 (1.5) [1.1-1.9]	12/58 (20.7) [11.6-33.7]
Anosmia or ageusia	978/3972 (24.6) [23.2-26.0]	264/978 (27.0) [24.3-29.9]
Fever	1581/3972 (39.8) [38.2-41.3]	8/1581 (0.5) [0.2-1.0]
Asthenia	1832/3972 (46.1) [44.6-47.7]	378/1832 (20.6) [18.8-22.6]
Attention or concentration disorders	376/3972 (9.5) [8.6-10.4]	84/376 (22.3) [18.3-27.0]
Memory loss	172/3972 (4.3) [3.7-5.0]	70/175 (40.0) [32.8-47.7]
Sleep disorders	421/3972 (10.6) [9.7-11.6]	154/421 (36.6) [32.0-41.4]
Cutaneous disorders	97/3972 (2.4) [2.0-3.0]	21/97 (21.6) [14.2-31.4]
Nausea	322/3972 (8.1) [7.0-9.0]	24/322 (7.5) [4.9-11.0]
Diarrhea	419/3972 (10.5) [9.6-11.6]	20/419 (4.8) [3.0-7.4]

**Table 3.** Estimated Proportion of Participants With Symptoms During the Acute Phase of SARS-CoV-2 Infection Whose Symptoms Resolved at 1 Year

Symptom	Resolution at 1 y after acute phase, % (95% CI)^a^
All symptoms	89.9 (88.7-90.9)
Cough	99.7 (99.6-99.8)
Dyspnea	95.8 (94.6-96.8)
Thoracic pain	99.3 (98.9-99.6)
Palpitation	93.0 (89.3-95.8)
Articular pain	91.5 (88.7-93.8)
Myalgia	99.9 (99.8-99.9)
Headache	99.9 (99.9-99.9)
Anosmia or ageusia	94.7 (93.5-95.6)
Fever	100 (100-100)
Asthenia	97.5 (97.1-97.9)
Attention or concentration disorders	94.2 (92.2-96.0)
Memory loss	77.5 (69.8-84.8)
Sleep disorders	79.9 (75.6-84.3)
Nausea	99.9 (99.8-99.9)
Diarrhea	100 (100-100)

^a^ Confidence interval (bootstrap).

**Figure.**
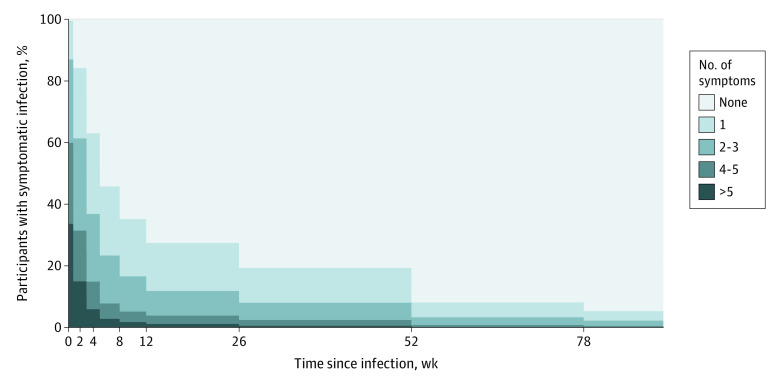
Distribution of the Number of Symptoms Depending on Time Since Acute Infection The number of individuals reporting the number of symptoms at each time step. For example, after 52 weeks, no participant reported having more than 5 symptoms.

Time to resolution of each symptom varied; an estimated 97.5% of patients with asthenia (95% CI, 97.1%-97.9%), 94.2% of patients with attention or concentration disorders ( 95% CI, 92.2%-96.0%), and 77.5% of patients with memory loss (95% CI, 69.8%-84.8%) experienced resolution of symptoms 1 year after the acute symptoms (Table 3; eFigure 3 in Supplement 1).

Older age (>60 years; HR, 0.78; 95% CI, 0.68-0.90), female sex (HR, 0.64; 95% CI, 0.58-0.70), history of cancer (HR, 0.61; 95% CI, 0.47-0.79), history of tobacco consumption (HR, 0.80; 95% CI, 0.73-0.88), high body mass index (BMI; calculated as weight in kilograms divided by height in meters squared) (≥30: HR, 0.75; 95% CI, 0.63-0.89), and high number of symptoms during the acute phase (>4; HR, 0.43; 95% CI, 0.39-0.48) were associated with a slower resolution of all symptoms (Table 4). Sensitivity analysis including all symptoms reported after the acute phase whatever their date of occurrence gave similar results (eTable 5 in Supplement 1). Analysis of factors associated with symptom resolution in the 2 subgroups defined by the mode of diagnosis of SARS-CoV-2 infection (serologic test or PCR test) did not change the main findings (eTable 6 in Supplement 1). These factors and other comorbid conditions were also not consistently associated with some specific symptoms; female sex was associated with a slower resolution of anosmia or ageusia, while older age, female sex, history of anxiety or depression, history of cancer, history of diabetes, tobacco consumption, high BMI, and high number of acute symptoms were associated with a slower resolution of asthenia. Slower resolution of attention or concentration disorders was associated with older age only (eTable 4 in Supplement 1).

**Table 4.** Factors Associated With Overall Symptom Resolution

Variable	HR (95% CI)	*P* value	aHR (95% CI)	*P* value
Age, y (reference: ≤40 y)				
40 to ≤60	0.87 (0.78-0.98)	.02	0.88 (0.78-0.99)	.03
>60	0.78 (0.68-0.90)	<.001	0.79 (0.67-0.92)	.003
Female (reference: male)	0.64 (0.58-0.70)	<.001	0.67 (0.61-0.75)	<.001
Anxiety or depression	0.51 (0.35-0.73)	<.001	0.73 (0.51-1.06)	.12
Chronic respiratory disease	0.60 (0.47-0.75)	<.001	0.83 (0.65-1.05)	.10
Cancer	0.61 (0.47-0.79)	<.01	0.68 (0.52-0.90)	.007
Hypertension	0.75 (0.60-0.94)	.01	0.94 (0.74-1.18)	.57
Diabetes	0.55 (0.35-0.86)	.008	0.76 (0.46-1.28)	.30
Chronic cardiologic disorder	0.76 (0.49-1.20)	.25	0.84 (0.52-1.35)	.47
History of tobacco consumption	0.80 (0.73-0.88)	<.001	0.87 (0.79-0.97)	.88
BMI (reference: <25)				
25 to <30	0.86 (0.77-0.97)	.01	0.85 (0.75-0.96)	.01
≥30	0.75 (0.63-0.89)	<.001	0.82 (0.68-0.97)	.03
No. of acute symptoms (reference: ≤4)	0.43 (0.39-0.48)	<.001	0.45 (0.41-0.50)	<.001

Abbreviations: aHR, adjusted hazard ratio; BMI, body mass index (calculated as weight in kilograms divided by height in meters squared); HR, hazard ratio.

## Discussion

The temporal dynamics of symptoms after acute SARS-CoV-2 infection showed a rapid decrease during the first 6 months. However, approximately 10% of people with symptoms still presented with at least 1 symptom at 1 year after infection. Up to now, studies on the persistence of symptoms did not exceed 8 to 10 months of follow-up and were performed mainly in specific populations, including individuals entering care for COVID-19 management or who were selected because they had persistent symptoms.^4,13,14,15^ Nevertheless, our results are in line with another recent study examining persistent symptoms within the general population in England but with a shorter follow-up.^7^

The factors associated with a slower resolution of all symptoms were older age, female sex, history of cancer, history of tobacco consumption, higher BMI, and higher number of symptoms during the acute phase.^4,13^ These factors, but also other comorbid conditions, were associated with the risk of persistence of specific symptoms. Altogether, these findings suggest the need to optimally manage comorbid conditions in individuals with long COVID to help reduce the duration of their symptoms.

### Limitations

This work has some limitations. First, it focuses on symptoms reported during the acute phase of the disease because their association with the infectious event is more straightforward. We may have missed some participants with no symptoms during the acute phase but with symptom onset within 3 months of infection, as defined by the WHO. However, a sensitivity analysis considering all symptoms whatever their date of occurrence did not change our main results (eTable 5 in Supplement 1). Second, detailed information on symptom durations was collected in the follow-up questionnaire, and recall bias cannot be excluded. This factor may lead to an underestimation of the rate at which symptoms resolved during the first few months. Third, the symptoms described may not be directly associated with SARS-CoV-2 infection. However, we reduced the risk of this possibility by considering only symptoms that occurred at the time or within 2 weeks of acute infection. Fourth, only participants with internet access were invited to participate, and only a fraction of them did participate. Thus, the population is not representative of the general French population. However, our objectives were not to provide nationally representative prevalence estimates of long COVID but rather to explore the duration of symptoms and the factors associated with their resolution among participants who reported SARS-CoV-2 infection. If selection bias were to occur, it can be assumed that participants with SARS-CoV-2 infection with few or short-lived symptoms might be less likely to participate than participants with persistent symptoms at the time of the questionnaire. Under this assumption, the durations of persistent symptoms in our study may be overestimated. Fifth, the WHO definition of the post–COVID-19 condition entails both the exclusion of an alternative diagnosis and its effect on everyday functioning. Misclassification may have occurred, and the proportion of participants with persistent symptoms associated with SARS-CoV-2 infection may be overestimated.

## Conclusions

This cross-sectional study found that most symptoms after SARS-CoV-2 infection disappear within 1 year. However, in a pandemic context with a high level of cumulative incidence, the absolute prevalent number of people with persistent symptoms after 1 year remains a public health concern.

## Notes

Supplement 1. **eFigure 1.** Flowchart of the Study **eTable 1.** Characteristics of Participants With Biological Result for SARS-CoV-2 Infection Stratified by Sex **eTable 2.** Characteristics of Participants With Biological Result for SARS-CoV-2 Infection Stratified by Age **eTable 3.** Characteristics of Participants With Positive Biological Result for SARS-CoV-2 Infection **eFigure 2.** Estimates of Survival for All Symptoms Resolution **eFigure 3.** Estimates of Survival for All Symptoms Resolution by Categories of Associated Factors **eTable 4.** Hazard Ratio for Each Symptom Resolution **eTable 5.** Sensitivity Analysis: Hazard Ratio for All Symptoms Resolution That Were Reported During the Follow-up **eTable 6.** Sensitivity Analysis: Hazard Ratio for All Symptoms Resolution in the Overall Population and in the Subpopulation Defined by a Positive Serology

Supplement 2. **Nonauthor Collaborators.** Santé, Pratiques, Relations et Inégalités Sociales en Population Générale Pendant la Crise COVID-19–Sérologie (SAPRIS-SERO) Study Group Members
