# Supplementary material for: Long-lasting Symptoms After an Acute COVID-19 Infection and Factors Associated With Their Resolution
Source: JAMA Netw Open. 2022 Nov 9;5(11):e2240985. doi: 10.1001/jamanetworkopen.2022.40985 (PMC9647489; doi:10.1001/jamanetworkopen.2022.40985)
Supplement: Supplement 1. — eFigure 1. Flowchart of the Study eTable 1. Characteristics of Participants With Biological Result for SARS-CoV-2 Infection Stratified by Sex eTable 2. Characteristics of Participants With Biological Result for SARS-CoV-2 Infection Stratified by Age eTable 3. Characteristics of Participants With Positive Biological Result for SARS-CoV-2 Infection eFigure 2. Estimates of Survival for All Symptoms Resolution eFigure 3. Estimates of Survival for All Symptoms Resolution by Categories of Associated Factors eTable 4. Hazard Ratio for Each Symptom Resolution eTable 5. Sensitivity Analysis: Hazard Ratio for All Symptoms Resolution That Were Reported During the Follow-up eTable 6. Sensitivity Analysis: Hazard Ratio for All Symptoms Resolution in the Overall Population and in the Subpopulation Defined by a Positive Serology [file jamanetwopen-e2240985-s001.pdf]

Supplementary Online Content

Robineau O, Zins M, Touvier M, et al; Santé, Pratiques, Relations et Inégalités Sociales en Population Générale Pendant la Crise COVID-19–Sérologie (SAPRIS-SERO) Study Group. Long-lasting symptoms after an acute COVID-19 infection and factors associated with their resolution. *JAMA Netw Open.* 2022;5(11):e2240985. doi:10.1001/jamanetworkopen.2022.40985

eFigure 1. Flowchart of the Study
eTable 1. Characteristics of Participants With Biological Result for SARS-CoV-2 Infection Stratified by Sex
eTable 2. Characteristics of Participants With Biological Result for SARS-CoV-2 Infection Stratified by Age
eTable 3. Characteristics of Participants With Positive Biological Result for SARS-CoV-2 Infection
eFigure 2. Estimates of Survival for All Symptoms Resolution
eFigure 3. Estimates of Survival for All Symptoms Resolution by Categories of Associated Factors
eTable 4. Hazard Ratio for Each Symptom Resolution
eTable 5. Sensitivity Analysis: Hazard Ratio for All Symptoms Resolution That Were Reported During the Follow-up
eTable 6. Sensitivity Analysis: Hazard Ratio for All Symptoms Resolution in the Overall Population and in the Subpopulation Defined by a Positive Serology

This supplementary material has been provided by the authors to give readers additional information about their work.

© 2022 Robineau O et al. *JAMA Network Open.*

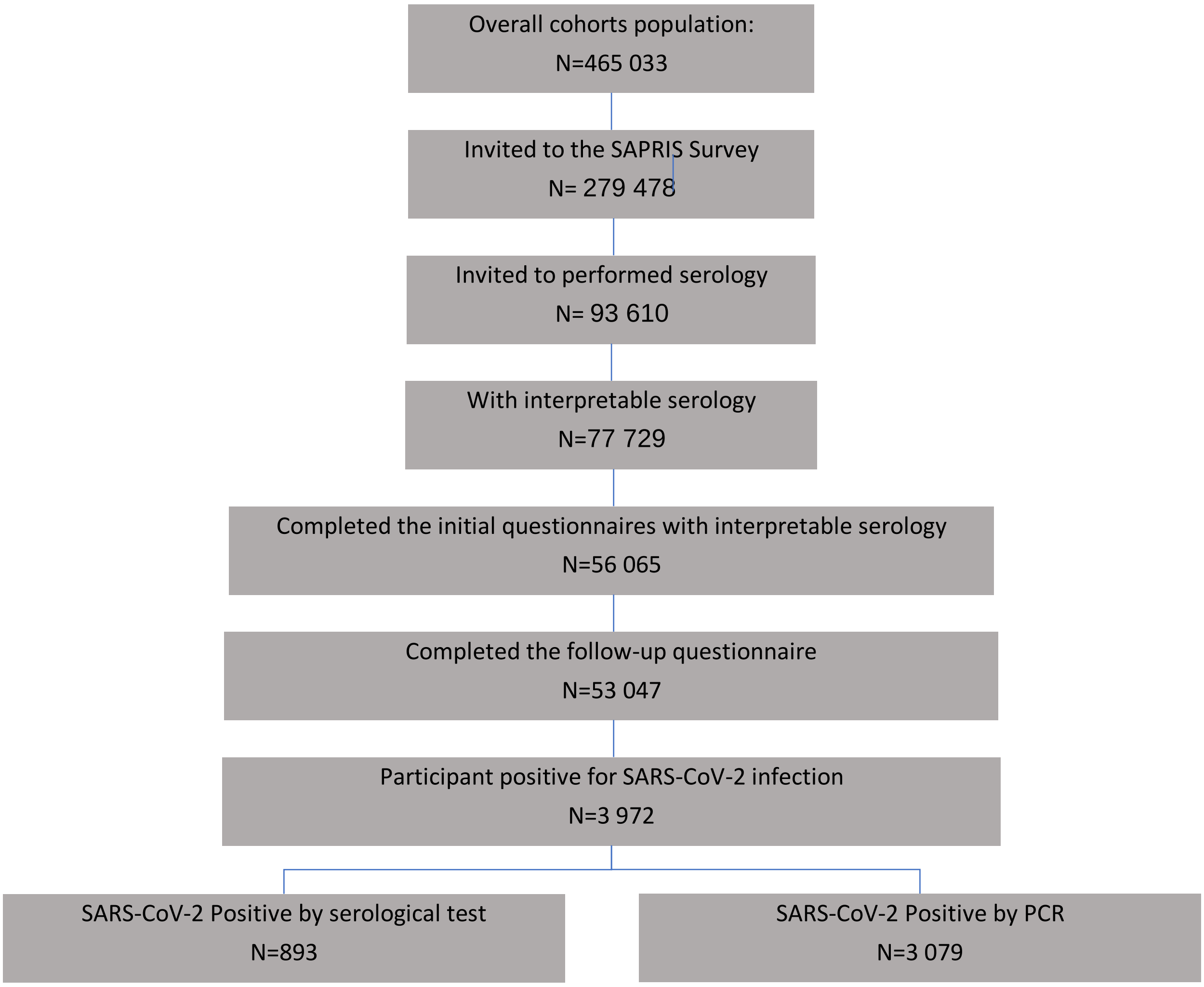

eFigure 1. Flowchart of the Study.

© 2022 Robineau O et al. *JAMA Network Open.*

	Male	Female	p
Variables	N= 1441 (%)	N=2531 (%)	
Age (SD)	52.0 (13.2)	50.2 (12.4)	<0.001
Chronic respiratory disease	54 (3.76)	204 (8.07)	<0.001
Anxiety/depression	12 (0.84)	82 (3.24)	<0.001
Cancer	49 (3.41)	114 (4.51)	0.11
HTA	108 (7.53)	156 (6.17)	0.11
Diabetes mellitus	29 (2.02)	44 (1.74)	0.61
Chronic cardiological disorder	51 (3.55)	20 (0.79)	<0.001
BMI (Kg/m^2) (SD)	25.3 (3.95)	23.9 (4.46)	<0.001
History of tobacco consumption	708 (50.5)	1203 (48.8)	0.32
Number of symptoms at the acute phase (SD)	2.67 (2.92)	3.27 (3.26)	<0.001
Persistent symptoms	212 (14.7)	649 (25.6)	<0.001

eTable 1. Characteristics of Participants With Biological Result for SARS-CoV-2 Infection Stratified by Sex.

© 2022 Robineau O et al. *JAMA Network Open.*

	Age<=40	40<Age>=60	Age>60	p
Variables	N=897 (%)	N=2066 (%)	N=1009 (%)	
Sex	593 (66.1)	1352 (65.4)	586 (58.1)	<0.001
Chronic respiratory disease	39 (4.35)	106 (5.14)	113 (11.3)	<0.001
Anxiety/depression	20 (2.23)	56 (2.71)	18 (1.79)	0.276
Cancer	22 (2.46)	64 (3.10)	77 (7.67)	<0.001
HTA	4 (0.45)	99 (4.80)	161 (16.0)	<0.001
Diabetes mellitus	2 (0.22)	19 (0.92)	52 (5.18)	<0.001
Chronic cardiological disorder	0 (0.00)	22 (1.07)	49 (4.88)	<0.001
History of tobacco consumption	362 (41.3)	963 (47.9)	586 (59.8)	<0.001
BMI (Kg/m^2) (SD)	23.5 (4.47)	24.5 (4.35)	24.9 (4.07)	<0.001
Number of symptoms at the acute phase (SD)	3.40 (3.31)	3.18 (3.13)	2.50 (2.99)	<0.001
Persistent symptoms	169 (18.9)	474 (22.9)	218 (21.6)	0.05

eTable 2. Characteristics of Participants with Biological Result for SARS-CoV-2 Infection Stratified by Age.

© 2022 Robineau O et al. *JAMA Network Open.*

	**Asymptomatic infection N=1324**	**Symptomatic infection N=2648**	**p**
**Age**			<0.001
**<40**	273 (20.6%)	624 (23.6%)	
40>age>=60	641 (48.4%)	1425 (53.8%)	
>60	410 (31.0%)	599 (22.6%)	
**Sex**			0.041
male	510 (38.5%)	931 (35.2%)	
female	814 (61.5%)	1717 (64.8%)	
**Anxiety/depression**			0.68
No	1293 (97.8%)	2576 (97.5%)	
Yes	29 (2.19%)	65 (2.46%)	
**Chronic respiratory disease**			0.38
No	1229 (93.0%)	2476 (93.8%)	
Yes	93 (7.03%)	165 (6.25%)	
**Cancer**			0.88
No	1269 (96.0%)	2531 (95.8%)	
Yes	53 (4.01%)	110 (4.17%)	
**HTA**			0.002
No	1211 (91.6%)	2488 (94.2%)	
Yes	111 (8.40%)	153 (5.79%)	
**Diabetes mellitus**			0.59
No	1295 (98.0%)	2595 (98.3%)	
Yes	27 (2.04%)	46 (1.74%)	
**Chronic cardiological disorder**			0.22
No	1293 (97.8%)	2599 (98.4%)	
Yes	29 (2.19%)	42 (1.59%)	
**History of tobacco consomption**			0.61
No	655 (51.2%)	1302 (50.3%)	
Yes	624 (48.8%)	1287 (49.7%)	
**BMI**			0.58
<=25	817 (63.4%)	1651 (64.3%)	
25<BMI<=30	351 (27.3%)	662 (25.8%)	
BMI>=30	120 (9.32%)	254 (9.89%)	
**Number of acute symptoms**			-
<=4	-	1467 (55.4%)	
>4	-	1181 (44.6%)	

eTable 3. Characteristics of Participants with Positive Biological Result for SARS-CoV-2 Infection.

© 2022 Robineau O et al. *JAMA Network Open.*

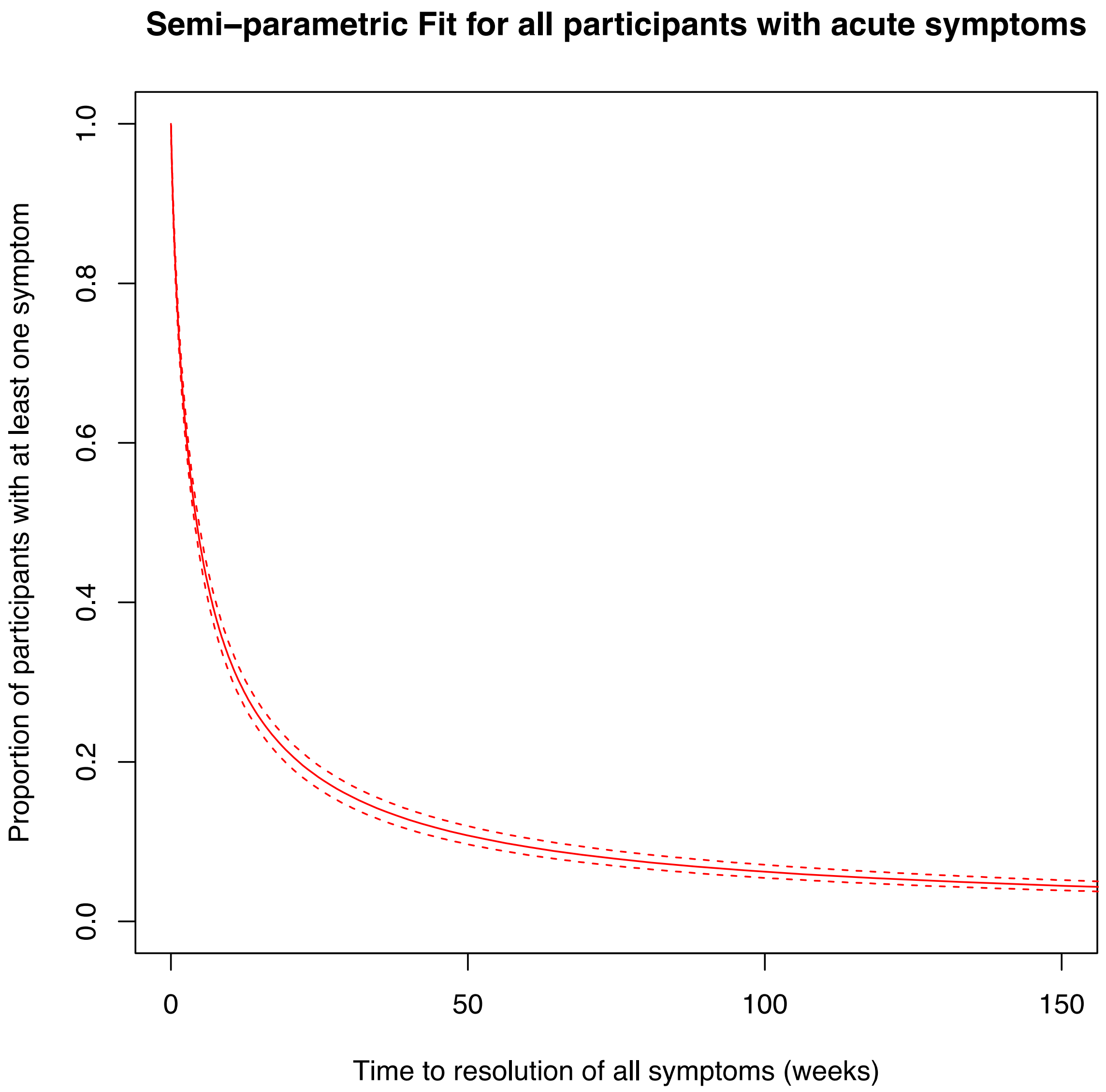

eFigure 2. Estimates of Survival for All Symptoms Resolution.

© 2022 Robineau O et al. *JAMA Network Open.*

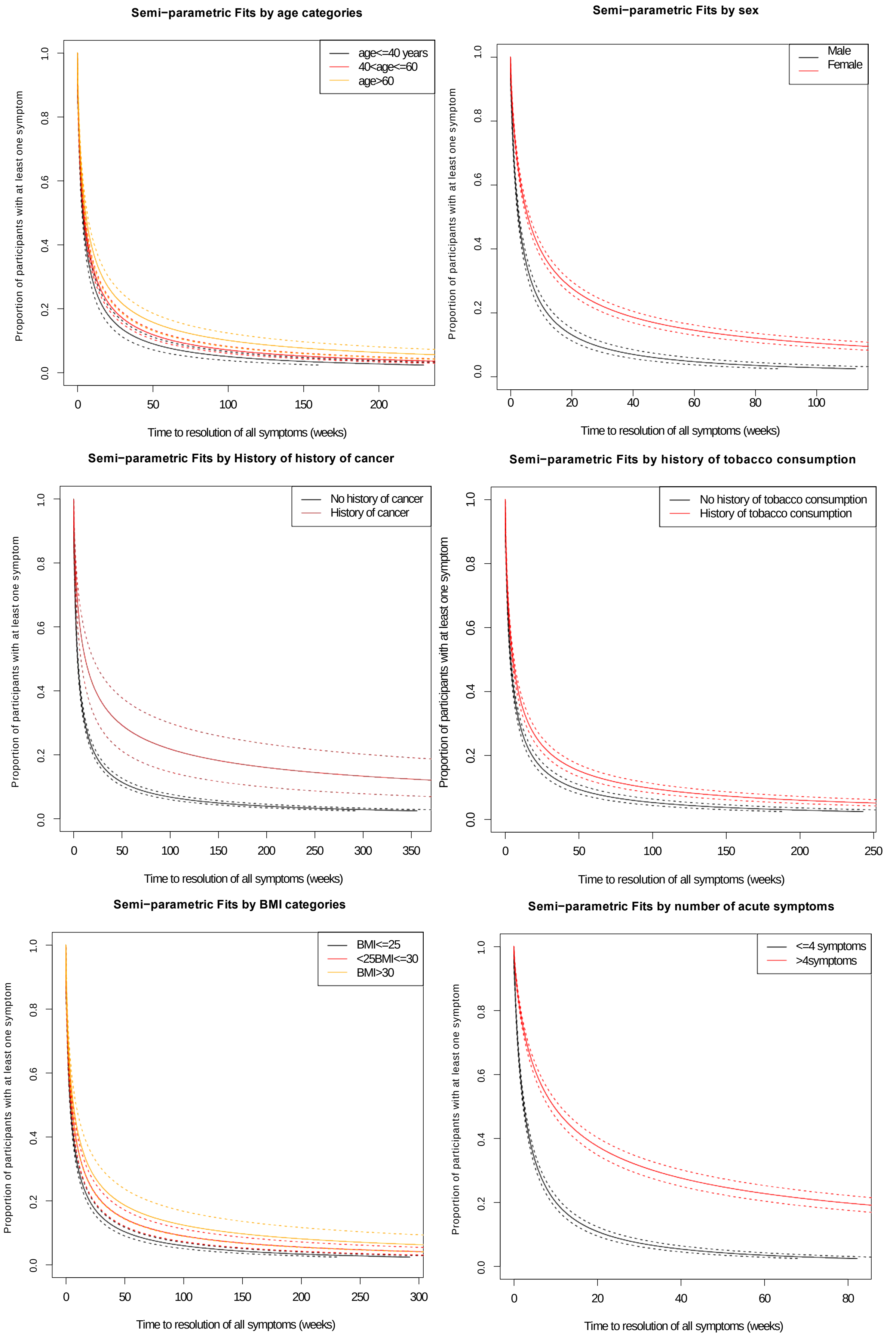

eFigure 3. Estimates of Survival for All Symptoms Resolution by Categories of Associated Factors.

© 2022 Robineau O et al. *JAMA Network Open.*

Variables	Cough aHR [95%CI]	Dyspnea aHR [95%CI]	Thoracic pain aHR [95%CI]	Palpitation aHR [95%CI]	Articular pain aHR [95%CI]	Myalgia aHR [95%CI]
**(base=<40)**						
40>age>=60	**0.71 [0.61-0.83]**	0.91 [0.71-1.15]	0.81 [0.6-1.1]	0.87 [0.53-1.44]	**0.67 [0.49-0.93]**	**0.75 [0.65-0.87]**
>60	**0.55 [0.45-0.67]**	0.82 [0.6-1.13]	0.87 [0.59-1.3]	0.62 [0.31-1.25]	**0.43 [0.28-0.66]**	**0.63 [0.52-0.75]**
**Sex (base= male)**	**0.84 [0.73-0.96]**	**0.70 [0.57-0.87]**	0.90 [0.7-1.17]	0.96 [0.55-1.68]	0.82 [0.61-1.1]	0.92 [0.81-1.05]
**Anxiety/depression**	1.21 [0.76-1.92]	0.66 [0.36-1.19]	0.93 [0.43-2.01]	1.39 [0.66-2.91]	0.58 [0.25-1.3]	0.77 [0.5-1.18]
**Chronic respiratory disease**	0.84 [0.62-1.13]	0.82 [0.56-1.21]	1.31 [0.79-2.17]	0.92 [0.37-2.31]	0.7 [0.4-1.23]	0.92 [0.68-1.25]
**History of cancer**	1.22 [0.79-1.87]	0.76 [0.46-1.26]	0.69 [0.42-1.14]	0.54 [0.07-3.94]	0.62 [0.29-1.3]	**0.69 [0.49-0.97]**
**HTA**	1.01 [0.73-1.4]	0.98 [0.6-1.61]	0.89 [0.08-9.91]	0.62 [0.12-3.25]	1.19 [0.67-2.13]	0.88 [0.68-1.13]
**Diabetes mellitus**	0.68 [0.38-1.21]	0.83 [0.34-2.01]	-	1.43 [0.03-58.69]	0.59 [0.01-59.94]	0.53 [0.05-5.63]
**Chronic cardiological disorder**	0.76 [0.44-1.31]	0.76 [0.41-1.41]	-	0.82 [0-487.33]	1.34 [0.03-67.61]	0.79 [0.5-1.25]
**History of tobacco consumption**	0.96 [0.84-1.1]	0.95 [0.78-1.15]	1.03 [0.81-1.3]	0.98 [0.64-1.52]	0.91 [0.7-1.18]	0.96 [0.85-1.08]
**BMI (base: <=25)**						
25<BMI<=30	0.92 [0.78-1.07]	0.82 [0.64-1.05]	0.91 [0.68-1.22]	0.79 [0.46-1.35]	1.07 [0.78-1.47]	0.90 [0.78-1.04]
BMI>=30	**0.77 [0.61-0.96]**	0.77 [0.56-1.05]	0.74 [0.47-1.18]	1.08 [0.58-2.02]	0.72 [0.5-1.03]	**0.73 [0.59-0.89]**
**Number of acute symptoms (base=<=4)**	**0.84 [0.73-0.96]**	1.12 [0.88-1.42]	0.79 [0.56-1.11]	1.29 [0.65-2.55]	1.11 [0.75-1.65]	**0.74 [0.65-0.85]**

Variables	Headache aHR [95%CI]	Anosmia/ageusia aHR [95%CI]	Fever aHR [95%CI]	Diarrhoae aHR [95%CI]	Nausea aHR [95%CI]
**age (base: <=40 years)**					
40>age>=60	0.94 [0.82-1.07]	1.05 [0.88-1.25]	**0.78 [0.68-0.89]**	0.86 [0.64-1.17]	0.79 [0.57-1.08]
>60	0.89 [0.75-1.07]	0.96 [0.75-1.21]	**0.63 [0.52-0.75]**	0.96 [0.67-1.37]	0.72 [0.49-1.06]
**Sex (base= male)**	**0.80 [0.7-0.91]**	**0.77 [0.66-0.91]**	1.04 [0.92-1.16]	0.89 [0.68-1.17]	0.71 [0.51-1]
**anxiety/depression**	0.89 [0.66-1.21]	0.86 [0.49-1.51]	**0.67 [0.48-0.94]**	0.53 [0.25-1.14]	0.67 [0.39-1.12]
**Chronic respiratory disease**	0.84 [0.65-1.07]	0.98 [0.65-1.47]	1.10 [0.86-1.4]	0.79 [0.49-1.28]	1.01 [0.58-1.75]
**History of cancer**	0.95 [0.7-1.28]	0.94 [0.57-1.55]	0.77 [0.57-1.04]	1.04 [0.63-1.71]	0.74 [0.39-1.39]
**HTA**	0.98 [0.76-1.25]	0.89 [0.57-1.39]	0.93 [0.73-1.19]	0.83 [0.5-1.39]	0.93 [0.49-1.78]
**Diabetes mellitus**	1.10 [0.56-2.15]	1.53 [0.01-259.9]	1.38 [0.92-2.09]	-	-
**Chronic cardiological disorder**	0.92 [0.55-1.55]	-	0.73 [0.49-1.07]	-	-
**History of tobaco consumption**	0.90 [0.81-1.01]	0.90 [0.78-1.05]	0.97 [0.86-1.09]	0.97 [0.77-1.23]	0.86 [0.64-1.17]
**BMI (base: <=25)**					
25<BMI<=30	**0.88 [0.77-0.99]**	0.89 [0.74-1.06]	**0.83 [0.73-0.95]**	0.85 [0.64-1.13]	0.90 [0.65-1.25]
BMI>=30	**0.74 [0.61-0.9]**	0.85 [0.63-1.15]	**0.66 [0.54-0.79]**	0.95 [0.68-1.32]	1.10 [0.72-1.66]
**Number of acute symptoms (base=<=4)**	**0.84 [0.75-0.95]**	1.06 [0.91-1.24]	**0.80 [0.71-0.9]**	1.09 [0.81-1.47]	1.13 [0.8-1.6]

eTable 4. Hazard Ratio for Each Symptom Resolution. **aHR: Hazard Ratio; CI: confidence interval. Results with p<0.05 are in bold.**

© 2022 Robineau O et al. *JAMA Network Open.*

Variables	Asthenia aHR [95%CI]	Attention/ Concentration disorders aHR [95%CI]	Memory loss aHR [95%CI]	Sleep disorder aHR [95%CI]
**age (base: <=40 years)**				
40>age>=60	**0.69 [0.61-0.79]**	0.76 [0.57-1.03]	1.04 [0.55-1.98]	0.83 [0.58-1.17]
>60	**0.65 [0.55-0.77]**	0.56 [0.38-0.83]	0.42 [0.16-1.07]	0.86 [0.58-1.26]
**Sex (base= male)**	**0.65 [0.58-0.73]**	0.76 [0.57-1.01]	0.62 [0.31-1.22]	**0.6 [0.43-0.82]**
**anxiety/depression**	**0.52 [0.34-0.8]**	0.48 [0.22-1.06]	-	0.47 [0.1-2.21]
**Chronic respiratory disease**	0.86 [0.66-1.13]	0.93 [0.48-1.79]	-	1.19 [0.66-2.14]
**History of cancer**	**0.61 [0.44-0.84]**	0.50 [0.25-1.03]	-	0.73 [0.32-1.67]
**HTA**	0.96 [0.74-1.24]	1.71 [0.7-4.19]	-	1.89 [0.98-3.65]
**Diabetes mellitus**	**0.57 [0.33-0.98]**	-	-	0.69 [0.01-52.92]
**Chronic cardiological disorder**	0.94 [0.59-1.48]	-	-	1.05 [0.1-11.4]
**History of tobacco consumption**	**0.88 [0.79-0.99]**	1.06 [0.81-1.38]	1.26 [0.8-1.98]	**0.74 [0.55-0.99]**
**BMI (base: <=25)**				
25<BMI<=30	**0.85 [0.74-0.97]**	1.20 [0.87-1.66]	0.80 [0.39-1.62]	0.76 [0.54-1.07]
BMI>=30	**0.76 [0.63-0.93]**	1.13 [0.71-1.81]	1.49 [0.88-2.51]	0.68 [0.41-1.1]
**Number of acute symptoms (base=<=4)**	**0.58 [0.51-0.65]**	0.95 [0.57-1.59]	0.65 [0.37-1.16]	1.65 [1.13-2.41]

eTable 4. Hazard Ratio for Each Symptom Resolution. **aHR: Hazard Ratio; CI: confidence interval. Results with p<0.05 are in bold**

© 2022 Robineau O et al. *JAMA Network Open.*

Variables	aHR (%95%)	p
**Age (base: <=40 years)**		
40>age>=60	0.8 [0.71-0.9]	<0.001
>60	0.78 [0.67-0.91]	0.001
**Sex (base= male)**	0.64 [0.58-0.71]	<0.001
**Anxiety/depression**	0.61 [0.4-0.92]	0.02
**Chronic respiratory disease**	0.95 [0.75-1.2]	0.66
**Cancer**	0.65 [0.49-0.86]	0.003
**HTA**	0.97 [0.75-1.24]	0.78
**Diabetes mellitus**	0.71 [0.41-1.21]	0.21
**Chronic cardiological disorder**	0.77 [0.49-1.22]	0.27
**History of tobacco consomption**	0.85 [0.77-0.94]	0.001
**BMI (base: <=25)**		
25<BMI<=30	0.86 [0.76-0.97]	0.01
BMI>=30	0.78 [0.65-0.95]	0.10
**Number of acute symptoms (base=<=4)**	0.52 [0.47-0.58]	<0.001

eTable 5. Sensitivity Analysis: Hazard Ratio for All Symptoms Resolution That Were Reported During the Follow-up. aHR: adjusted Hazard Ratio; CI: confidence interval. 2835 (71.4%) individuals with a positive biological test result declared at least one symptom during the follow-up.

© 2022 Robineau O et al. *JAMA Network Open.*

Variables	Results of the survey (PCR + serology) aHR [95%CI]	p	Sensitivity analysis (serology only) aHR [95%CI]	p
**Age**				
40>age>=60	0.88 [0.78-0.99]	0.03	0.55 [0.33-1.15]	0.12
>60	0.79 [0.67-0.92]	0.003	0.43 [0.19-0.92]	0.01
**Sex (base= male)**	0.67 [0.61-0.75]	<0.001	0.80 [0.48-1.20]	0.31
**Anxiety/depression**	0.73 [0.51-1.06]	0.12	-	-
**Chronic respiratory disease**	0.83 [0.65-1.05]	0.10	0.22 [0.10-0.98]	0.03
**Cancer**	0.68 [0.52-0.90]	0.007		
**HTA**	0.94 [0.74-1.18]	0.57	0.64 [0.25-1.83]	0.64
**Diabetes mellitus**	0.76 [0.46-1.28]	0.30	-	-
**Chronic cardiological disorder**	0.84 [0.52-1.35]	0.47	-	-
**History of tobacco consumption**	0.87 [0.79-0.97]	0.88	0.92 [0.52-1.59]	0.94
**BMI (base: <=25)**				
25<BMI<=30	0.85 [0.75-0.96]	0.01	0.47 [0.25-0.90]	0.003
BMI>=30	0.82 [0.68-0.97]	0.03	0.55 [0.26-0.95]	0.03
**Number of acute symptoms (base=<=4)**	0.45 [0.41-0.5]	<0.001	0.90 [0.47-0.1.10]	0.56

eTable 6. Sensitivity Analysis: Hazard Ratio for All Symptoms Resolution in the Overall Population and in the Subpopulation Defined by a Positive Serology

© 2022 Robineau O et al. *JAMA Network Open.*
