# Supplementary material for: Long-lasting Symptoms After an Acute COVID-19 Infection and Factors Associated With Their Resolution
Source: JAMA Netw Open. 2022 Nov 9;5(11):e2240985. doi: 10.1001/jamanetworkopen.2022.40985 (PMC9647489; doi:10.1001/jamanetworkopen.2022.40985)
Supplement: Supplement 2. — Nonauthor Collaborators. Santé, Pratiques, Relations et Inégalités Sociales en Population Générale Pendant la Crise COVID-19–Sérologie (SAPRIS-SERO) Study Group Members [file jamanetwopen-e2240985-s002.pdf]

Supplemental Online Content: Nonauthor Collaborators
*First name, last name, and suffix (if applicable) are required and will appear in PubMed.

***Group Name(s): Santé, Pratiques, Relations et Inégalités Sociales en Population Générale Pendant la Crise COVID-19–Sérologie (SAPRIS-SERO) Study Group**							
*First Name and Middle Initial(s)	*Last Name	***Suffix (eg, Jr, III)**	Academic Degrees	Institution	Location (city, state/province, country)	Role or Contribution, eg, chair, principal investigator	Group (if more than 1 Group listed in the byline) and/or Subgroup (eg, Steering Committee)
Fabrice	Carrat		PhD	Institut Pierre-Louis d'Épidémiologie et de Santé Publique, Sorbonne Université, Inserm, Département de santé publique, Hôpital Saint-Antoine, APHP, 27 rue Chaligny, 75571 Paris Cedex 12, France	Paris	PI	
Marie	Zins		PhD	UVSQ, Inserm UMS 11, Université Paris-Saclay, Université de Paris, Villejuif, France	Villejuif		
Gianluca	Severi		PhD	CESP UMR1018, UVSQ, Inserm, Université Paris-Saclay, Gustave Roussy, Villejuif, France	Paris		
Mathilde	Touvier		PhD	nserm U1153, Inrae U1125, Cnam, Nutritional Epidemiology Research Team (EREN), Sorbonne Paris Nord University, Epidemiology and Statistics Research Center – University of Paris (CRESS), Bobigny, France	Paris		
Hélène	Blanché		PhD	Fondation Jean Dausset-CEPH (Centre d'Etude du Polymorphisme Humain), CEPH-Biobank, Paris, France	Paris		
Jean-François	Deleuze		PhD	Fondation Jean Dausset-CEPH (Centre d'Etude du Polymorphisme Humain), CEPH-Biobank, Paris, France	Paris		
Xavier	De Lambalerie		PhD	Unité des Virus Émergents, UVE, IRD 190, INSERM 1207, Aix Marseille Univ, IHU Méditerranée Infection, Marseille, France	Marseille		
Clovis	Lusivika-Nzinga			Institut Pierre-Louis d'Épidémiologie et de Santé Publique, Sorbonne Université, Inserm, Département de santé publique, Hôpital Saint-Antoine, APHP, 27 rue Chaligny, 75571 Paris Cedex 12, France	Paris		

Supplemental Online Content: Nonauthor Collaborators
*First name, last name, and suffix (if applicable) are required and will appear in PubMed.

*First Name and Middle Initial(s)	*Last Name	***Suffix (eg, Jr, III)**	Academic Degrees	Institution	Location (city, state/province, country)	Role or Contribution, eg, chair, principal investigator	Group (if more than 1 Group listed in the byline) and/or Subgroup (eg, Steering Committee)
Gregory	Pannetier		PhD	Institut Pierre-Louis d'Épidémiologie et de Santé Publique, Sorbonne Université, Inserm, Département de santé publique, Hôpital Saint-Antoine, APHP, 27 rue Chaligny, 75571 Paris Cedex 12, France	Paris		
Nathanael	Lapidus		PhD	nstitut Pierre-Louis d'Épidémiologie et de Santé Publique, Sorbonne Université, Inserm, Département de santé publique, Hôpital Saint-Antoine, APHP, 27 rue Chaligny, 75571 Paris Cedex 12, France	Paris		
Isabelle	Goderel			nstitut Pierre-Louis d'Épidémiologie et de Santé Publique, Sorbonne Université, Inserm, Département de santé publique, Hôpital Saint-Antoine, APHP, 27 rue Chaligny, 75571 Paris Cedex 12, France	Paris		
Céline	Dorival		PhD	nstitut Pierre-Louis d'Épidémiologie et de Santé Publique, Sorbonne Université, Inserm, Département de santé publique, Hôpital Saint-Antoine, APHP, 27 rue Chaligny, 75571 Paris Cedex 12, France	Paris		
Jerome	Nicol			nstitut Pierre-Louis d'Épidémiologie et de Santé Publique, Sorbonne Université, Inserm, Département de santé publique, Hôpital Saint-Antoine, APHP, 27 rue Chaligny, 75571 Paris Cedex 12, France	Paris		
Olviier	Robineau		PhD	nstitut Pierre-Louis d'Épidémiologie et de Santé Publique, Sorbonne Université, Inserm, Département de santé publique, Hôpital Saint-Antoine, APHP, 27 rue Chaligny, 75571 Paris Cedex 12, France	Paris		
Sofiane	Kab		PhD	UVSQ, Inserm UMS 11, Université Paris-Saclay, Université de Paris, Villejuif, France	Villejuif		

*First name, last name, and suffix (if applicable) are required and will appear in PubMed.

*First Name and Middle Initial(s)	*Last Name	***Suffix (eg, Jr, III)**	Academic Degrees	Institution	Location (city, state/province, country)	Role or Contribution, eg, chair, principal investigator	Group (if more than 1 Group listed in the byline) and/or Subgroup (eg, Steering Committee)
Adeline	Renuy			UVSQ, Inserm UMS 11, Université Paris-Saclay, Université de Paris, Villejuif, France	Villejuif		
Stéphane	Le-Got			UVSQ, Inserm UMS 11, Université Paris-Saclay, Université de Paris, Villejuif, France	Villejuif		
Céline	Ribet			UVSQ, Inserm UMS 11, Université Paris-Saclay, Université de Paris, Villejuif, France	Villejuif		
Miereille	Pellicer			UVSQ, Inserm UMS 11, Université Paris-Saclay, Université de Paris, Villejuif, France	Villejuif		
Emmanuel	Wiernik			UVSQ, Inserm UMS 11, Université Paris-Saclay, Université de Paris, Villejuif, France	Villejuif		
Marcel	Goldberg		PhD	UVSQ, Inserm UMS 11, Université Paris-Saclay, Université de Paris, Villejuif, France	Villejuif		
Fanny	Artaud			CESP UMR1018, UVSQ, Inserm, Université Paris-Saclay, Gustave Roussy, Villejuif, France	Villejuif		
Pascale	Gerbouin-			CESP UMR1018, UVSQ, Inserm, Université Paris-	Villejuif		
Mélodie	nguix			CESP UMR1018, UVSQ, Inserm, Université Paris-Saclay, Gustave Roussy, Villejuif, France	Villejuif		
Camille	Laplanche			CESP UMR1018, UVSQ, Inserm, Université Paris-Saclay, Gustave Roussy, Villejuif, France	Villejuif		
Roselyn	Gomes-Rima			CESP UMR1018, UVSQ, Inserm, Université Paris-Saclay, Gustave Roussy, Villejuif, France	Villejuif		
Lyan	Hoang			CESP UMR1018, UVSQ, Inserm, Université Paris-Saclay, Gustave Roussy, Villejuif, France	Villejuif		
Emmanuelle	Correia			CESP UMR1018, UVSQ, Inserm, Université Paris-Saclay, Gustave Roussy, Villejuif, France	Villejuif		
Alpha Amadou	Barry			CESP UMR1018, UVSQ, Inserm, Université Paris-Saclay, Gustave Roussy, Villejuif, France	Villejuif		
Nadège	Senina			CESP UMR1018, UVSQ, Inserm, Université Paris-Saclay, Gustave Roussy, Villejuif, France	Villejuif		

*First name, last name, and suffix (if applicable) are required and will appear in PubMed.

*First Name and Middle Initial(s)	*Last Name	***Suffix (eg, Jr, III)**	Academic Degrees	Institution	Location (city, state/province, country)	Role or Contribution, eg, chair, principal investigator	Group (if more than 1 Group listed in the byline) and/or Subgroup (eg, Steering Committee)
Julien	Allegre			nserm U1153, Inrae U1125, Cnam, Nutritional Epidemiology Research Team (EREN), Sorbonne Paris Nord University, Epidemiology and Statistics Research Center – University of Paris (CRESS), Bobigny, France	Bobigny		
Fabien	Szabo de Edelenyi			nserm U1153, Inrae U1125, Cnam, Nutritional Epidemiology Research Team (EREN), Sorbonne Paris Nord University, Epidemiology and Statistics Research Center – University of Paris (CRESS), Bobigny, France	Bobigny		
Nathalie	Druesne-Pecollo		PhD	nserm U1153, Inrae U1125, Cnam, Nutritional Epidemiology Research Team (EREN), Sorbonne Paris Nord University, Epidemiology and Statistics Research Center – University of Paris (CRESS), Bobigny, France	Bobigny		
Yunes	Esseddik		PhD	nserm U1153, Inrae U1125, Cnam, Nutritional Epidemiology Research Team (EREN), Sorbonne Paris Nord University, Epidemiology and Statistics Research Center – University of Paris (CRESS), Bobigny, France	Bobigny		
Serge	Hercberg			nserm U1153, Inrae U1125, Cnam, Nutritional Epidemiology Research Team (EREN), Sorbonne Paris Nord University, Epidemiology and Statistics Research Center – University of Paris (CRESS), Bobigny, France	Bobigny		
Mélanie	Deschasaux			nserm U1153, Inrae U1125, Cnam, Nutritional Epidemiology Research Team (EREN), Sorbonne Paris Nord University, Epidemiology and Statistics Research Center – University of Paris (CRESS), Bobigny, France	Bobigny		

*First name, last name, and suffix (if applicable) are required and will appear in PubMed.

*First Name and Middle Initial(s)	*Last Name	***Suffix (eg, Jr, III)**	Academic Degrees	Institution	Location (city, state/province, country)	Role or Contribution, eg, chair, principal investigator	Group (if more than 1 Group listed in the byline) and/or Subgroup (eg, Steering Committee)
Jean-Marc	Sébaoun			Fondation Jean Dausset-CEPH (Centre d'Etude du Polymorphisme Humain), CEPH-Biobank, Paris, France	Paris		
Jean-Christophe	Baudouin			Fondation Jean Dausset-CEPH (Centre d'Etude du Polymorphisme Humain), CEPH-Biobank, Paris, France	Paris		
Laetitia	Gressin			Fondation Jean Dausset-CEPH (Centre d'Etude du Polymorphisme Humain), CEPH-Biobank, Paris, France	Paris		
Valérie	Morel			Fondation Jean Dausset-CEPH (Centre d'Etude du Polymorphisme Humain), CEPH-Biobank, Paris, France	Paris		
Ouissam	Ouili			Fondation Jean Dausset-CEPH (Centre d'Etude du Polymorphisme Humain), CEPH-Biobank, Paris, France	Paris		
Laetitia	Ninove			Fondation Jean Dausset-CEPH (Centre d'Etude du Polymorphisme Humain), CEPH-Biobank, Paris, France	Paris		
Stéphane	Priet		PhD	Unité des Virus Émergents, UVE, IRD 190, INSERM 1207, Aix Marseille Univ, IHU Méditerranée Infection, Marseille, France	Marseille		
Paola Mariela	Saba Villarroel		PhD	Unité des Virus Émergents, UVE, IRD 190, INSERM 1207, Aix Marseille Univ, IHU Méditerranée Infection, Marseille, France	Marseille		
Toscane	Fourié		PhD	Unité des Virus Émergents, UVE, IRD 190, INSERM 1207, Aix Marseille Univ, IHU Méditerranée Infection, Marseille, France	Marseille		
Souand Mohamed	Ali		PhD	Unité des Virus Émergents, UVE, IRD 190, INSERM 1207, Aix Marseille Univ, IHU Méditerranée Infection, Marseille, France	Marseille		

Supplemental Online Content: Nonauthor Collaborators
*First name, last name, and suffix (if applicable) are required and will appear in PubMed.

*First Name and Middle Initial(s)	*Last Name	***Suffix (eg, Jr, III)**	Academic Degrees	Institution	Location (city, state/province, country)	Role or Contribution, eg, chair, principal investigator	Group (if more than 1 Group listed in the byline) and/or Subgroup (eg, Steering Committee)
Abdenour	Amroun		PhD	Unité des Virus Émergents, UVE, IRD 190, INSERM 1207, Aix Marseille Univ, IHU Méditerranée Infection, Marseille, France	Marseille		
Morgan	Seston		PhD	Unité des Virus Émergents, UVE, IRD 190, INSERM 1207, Aix Marseille Univ, IHU Méditerranée Infection, Marseille, France	Marseille		
Nazli	Ayhan		PhD	Unité des Virus Émergents, UVE, IRD 190, INSERM 1207, Aix Marseille Univ, IHU Méditerranée Infection, Marseille, France	Marseille		
Boris	Pastorino		PhD	Unité des Virus Émergents, UVE, IRD 190, INSERM 1207, Aix Marseille Univ, IHU Méditerranée Infection, Marseille, France	Marseille		
